# Quantum defects in two-dimensional van der Waals materials

**DOI:** 10.1016/j.fmre.2024.01.019

**Published:** 2024-02-08

**Authors:** Yang Guo, Jianmei Li, Ruifen Dou, Haitao Ye, Changzhi Gu

**Affiliations:** a Beijing National Laboratory for Condensed Matter Physics, Institute of Physics, Chinese Academy of Sciences, Beijing 100190, China; b School of Physical Sciences, CAS Key Laboratory of Vacuum Physics, University of Chinese Academy of Sciences, Beijing 100190, China; c Key Laboratory for Microstructural Material Physics of Hebei Province, School of Science, Yanshan University, Qinhuangdao 066004, China; d Department of Physics, Beijing Normal University, Beijing 100875, China; e Department of Engineering, University of Leicester, Leicester LE1 7RH, UK

**Keywords:** Quantum defect, Spin qubit, Single photon emission, Light-matter interaction, Defect engineering, Photonic structure, Quantum application

## Abstract

Quantum defects in solid materials, such as nitrogen-vacancy color centers in diamond, have been extensively studied and successfully demonstrated as single photon emitters and potential qubits for quantum computers. However, a major challenge has always been positioning these quantum defects near the sample surface for measuring or sensing purposes. The emergence of quantum defects in two-dimensional (2D) van der Waals (vdW) materials open up new opportunities for overcoming these limitations. These materials possess unique properties, including vdW interlayer coupling and clean surfaces without unsaturated dangling bonds, which provide greater advantages for manufacturing multi-qubit systems. In this review, we present the research progress on quantum defects in 2D vdW materials, covering quantum guidelines for spin defects in solid state, the latest demonstrations of quantum defects, the unique methods and techniques for generating and modulating defects in 2D vdW materials.

## 1 Introduction

Defects in the solid state play a critical role in condensed matter physics because of their strong ability to control and modulate material properties. Certain isolating point defects in the host materials behave like atoms with their own quantized levels, exhibiting quantum properties determined by their charge, spin states along with optical transitions. These defects, known as quantum defects, provide an ideal platform for exploiting the principles of quantum mechanics to enable advanced functionality and techniques beyond classical ones. The nitrogen-vacancy (*NV*) defect in diamond is a key representative of a quantum defect and has great potential for single photon emitters (SPEs) [1,2]. SPEs, as non-classical light sources [3], are essential for various quantum technologies, including quantum sensing [4,5], quantum communication [6] and quantum cryptography [7]. In addition, negative-charged *NV* (*NV*^−^) centers in diamond also have been identified as promising qubit candidates for quantum computers due to their optically addressable electron spin states and a long coherence time [1], hosting a photon-spin interface (PSI). Other color centers or quantum defects also have been observed in wide-gap semiconductors like silicon carbide and rare-earth-ion materials such as Er^3+^: CaWO_4_ [8,9]. These successful systems demonstrate high-fidelity initialization, manipulation, and measurement of qubits [10]. However, these quantum defects in bulk materials face inherent limitations originated from the three-dimensional (3D) nature of these materials. For instance, positioning these defects near the sample surface for measurements and sensing, as required by many schemes based on color centers, remains challenging. The spin coherence time of diamond *NV* centers sharply degrades in areas tens of nanometers from the surface [11,12], accompanied by decreased charge state stability and increased optical linewidths [13], [14], [15]. The electronic impurities present on the surface of bulk materials, resulting from uncontrolled dangling bonds and adsorbed contaminants, contribute to magnetic noise and spectral diffusion.

Recently discovered quantum defects in two-dimensional (2D) van der Waals (vdW) materials, including transition metal dichalcogenides (TMDs) and hexagonal boron nitride (h-BN), offer a new opportunity to overcome these limitations [16], [17], [18]. These 2D vdW materials exhibit reduced dimensionality and weak screening effects [19], leading to unique physical properties such as linear electron dispersion relation in graphene and strong excitonic effects in atomically thin TMDs flakes. h-BN, as a prominent 2D vdW material, hosts many atom-like defects that act as promising candidates for making SPEs and PSI [20], [21], [22]. The negatively-charged boron vacancy center ($V_{B}^{-}$) stands out as a well-understood quantum defect, exhibiting deterministic quantum emissions and spin-optical quantum properties for quantum applications [23]. In addition, theoretical predictions indicate that the spin-dependent lifetime of quantum defects on a monolayer TMD MoS_2_ can reach 30 ms [24], establishing it as an ideal material for manufacturing multiqubit operational computing systems. Compared to the 3D materials, 2D vdW materials possess advantages in achieving controllable generation and manipulation of qubits. With current experimental techniques, the defect location can be nearly deterministic in 2D vdW materials, presenting a new avenue for scaling up qubits of quantum computers.

This article reviews the recent advances of quantum defects in 2D vdW materials with a focus on implementing qubits. We start by highlighting quantum guidelines for spin defects in solid state in Section 2. The latest demonstrations of quantum defects in 2D vdW materials are introduced in Section 3, and the unique methods and techniques for generating and modulating defects in 2D vdW materials are presented in Section 4 and Section 5, respectively. The conclusion and outlook can be found in Section 6.

## 2 Quantum guidelines for spin defects in solid state

Defects in the solid state can effectively retain quantum information through their electron spin states, creating controllable qubits with prolonged spin relaxation ($T_{1}$) and coherence ($T_{2}$) times [19,20]. To achieve success in all quantum applications, it is crucial to optimize the fidelity of spin initialization, manipulation and readout. Therefore, spin states serve as the foundation of quantum hardware and have been the subject of extensive theoretical and experimental research. Identifying and designing new materials that host spin defects as qubits are essential steps in the development of quantum computers.

### 2.1 Physical criteria of materials hosting defect spin qubits

**Fig. 1:**
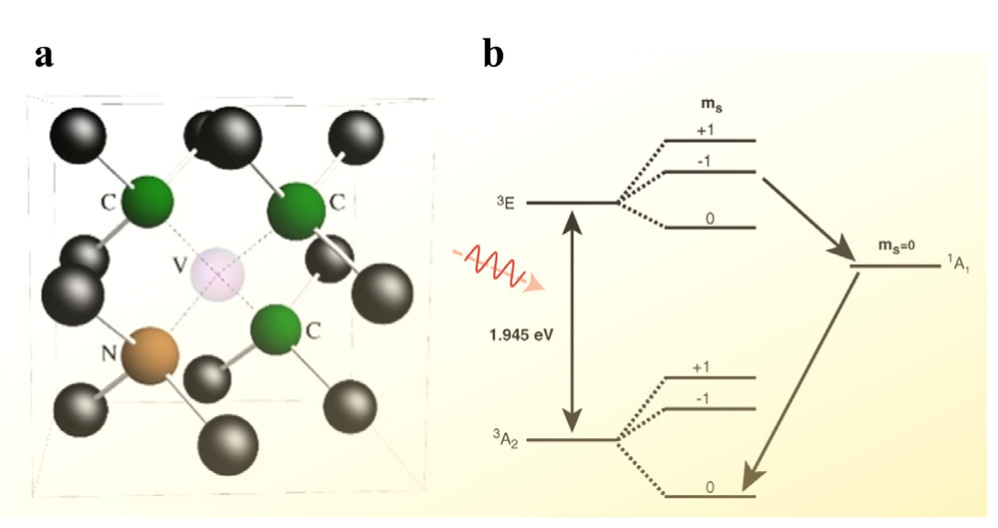
***NV*^−^ color centers in diamond**. (a) Schematic of *NV*^−^ in diamond lattice [1]. (b) Spin energy sublevels of the *NV*^−^ center [26].

To elucidate the basic principle of defect spin qubits, we first describe the electronic structures and optical transitions of spin states of a diamond *NV*^−^ center. As shown in Fig. 1a, the diamond *NV*^−^ center consists of a carbon vacancy and a nitrogen impurity that has substituted an adjacent atom. It contains six electrons, four of which originate from the atoms surrounding the vacancy, while the remaining two are from the bulk. The lowest energy bound state of the diamond *NV*^−^ center is a spin triplet (^3^A_2_), exhibiting a fine zero field splitting (ZFS) of approximately 2.88 GHz, which can be measured by conventional electron paramagnetic resonance (EPR). The *m_s_* = 0 and −1 spin sublevels of this ground state act as qubit states, with coherent rotations achievable by applying microwave wave with the frequency corresponding to the transition between them. In the absence of phonon interaction, the ^3^A_2_ ground state undergoes a spin-conserving optical transition to the ^3^*E* excited-state, known as a zero-phonon line with an energy of 1.945 eV. As shown in Fig. 1b, there exist an optical transition path between these two states, as well as a spin-selective nonradiative decay path between the spin-multiple states. It is evident that the *NV*^−^ center can be optically initialized and measured through these transitions, enabling optical excitation of the defect into the *m_s_* = 0 sublevel of ^3^A_2_ and facilitating the readout of two spin sublevels via their distinguishable luminescence.

Two characteristics of diamond *NV*^−^ centers distinguish them from other qubit systems in solid state: 1) the tightly confined bound states of defects are well decoupled from sources of decoherence; 2) they possess the capability to optically initialize and measure multiple spin states of the defect with great fidelity even in ambient conditions. Weber and Awschalom et al. have established generalized criteria that a quantum defect with spin qubits, as well as its host, must meet [26]. First, the quantum defects should exhibit the following five characteristics: (Q1) Qubit preparation: ability to prepare qubits with two or more spin sublevels using radio frequency waves; (Q2) Qubit initiation: an optical transition from the ground state to an excited state, accompanied by a spin-selective and nonradiative decay path between different spin multiple states; (Q3) Qubit readout: distinguishable luminescence of the qubit states, which varies depending on the qubit sublevel and should possess sufficient intensity, wavelength, or other property to facilitate efficient and high-fidelity measurement of single spin qubit states; (Q4) Qubit coherence: optical transitions without coupling to the electronic states of the host; (Q5) Qubit fidelity: bound states with adequate energy separations to prevent thermal excitation between them. Second, an ideal crystalline material should possess the following qualities: (M1) wide band gap to accommodate deep defects; (M2) weak spin-orbit coupling to avoid undesirable spin flips in the defect states; (M3) high-quality materials devoid of paramagnetic impurities that may impact the defect spin state; (M4) constituent elements with isotopes of zero nuclear spin. These requirements ensure long coherence time for qubits and minimize interactions between the spin and electric, thermal, and magnetic noise in the surroundings. These criteria have led to the discovery of various quantum materials as potential hosts for defect spin qubits, expanding the field beyond *NV*^−^ center in diamond and unveiling different materials with different advantages for defect spin qubits.

### 2.2 Spin relaxation and coherence of defects

**Fig. 2:**
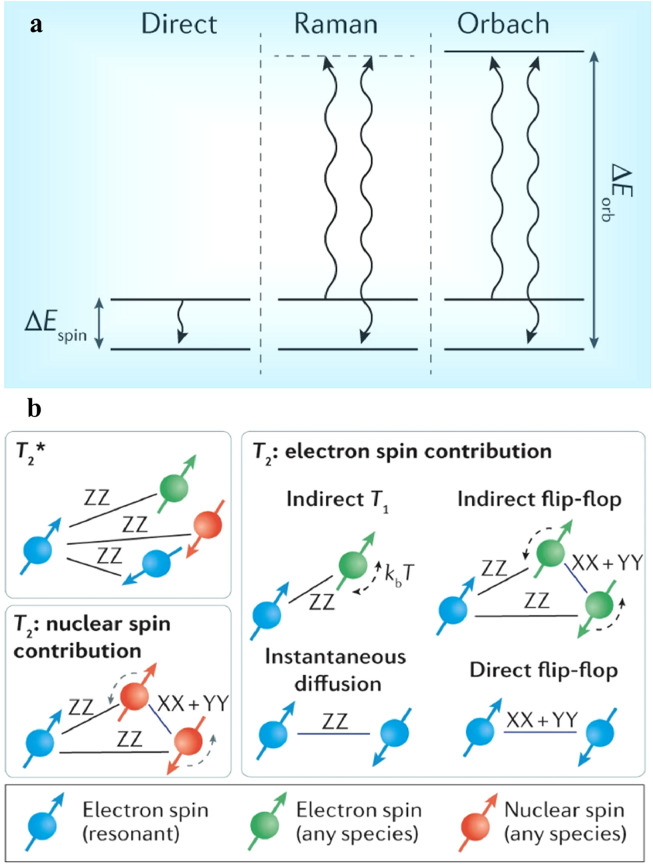
**Schematic of spin relaxation (a) and coherence (b)**[25]**.**

The spin relaxation time *T*_1_ is the characteristic time representing the dynamic process of transfer energy from spin to surrounding lattice through random spin flips. It fundamentally restricts the possible coherence time *T*_2_ [1,25]. Previous studies have shown that *T*_1_ is closely related to various parameters, such as lattice temperature, in many systems [27], [28], [29]. As illustrated in Fig. 2a, there are three typical mechanisms for spin-lattice relaxation [27]: resonant phonon absorption or emission between spin states, Raman processes and Orbach processes. The choice of Debye temperature in material design has a significant impact on the spin relaxation, particularly in the case of Raman processes. The Raman process is expected to have the following form ${\left(1/T_{1}\right)}_{\text{Raman}}\propto \;T^{5}$, while the Orbach process is expected to follow the form ${\left(1/T_{1}\right)}_{\text{Orbach}}\propto {\left(e^{\xi /kT}-1\right)}^{-1}$, where ξ is the most prominent local vibrational energy [29], [30], [31]. In addition, defects with highly spin-conserving orbital transitions can exhibit a long *T*_1_, even though phonon absorption and excitation are fast. The phonon bottleneck effect at cryogenic temperatures [32], where energy transfer is limited by slow phonon energy transfer, also slows down the spin relaxation process.

Furthermore, electron spin relaxation is not limited to phonon processes. For example, resonant electric or magnetic noise from the environment can effectively induce random spin flips [33,34]. External magnetic fields can also be used to tune spin relaxation by exploiting spin energy splitting mechanisms. Additionally, charge instability can cause a spin decay pathway, where the defect fluctuates between different charge states without preserving its spin [33]. Moreover, undesired photoexcitation to an excited state followed by a decay process can result in spin flips similar to those caused by phonons. Spin relaxation time *T*_1_ limited by phonon effects is one of the most difficult characteristics to improve for given conditions and materials. However, enhancing strain manipulation and designing a phonon bandgap can reduce the contribution from Orbach [30]. Spin relaxation time *T*_1_ of defect spins may theoretically be optimized with nanostructures shorter than the wavelength of pertinent acoustic phonons, although they may be affected by surface proximity effects [12], and the required scales typically exceed manufacturing capability [14]. At last, reducing $T_{1}$ is possible to achieve spin polarization through thermal relaxation, which can be accomplished by increasing the spontaneous emission in a microwave cavity [35].

Decoherence refers to the loss of phase information of quantum states. A long coherence time is essential to quantum application, allowing for longer phase acquisition times, longer memories, and higher control fidelities. Generally, surrounding fluctuating magnetic sources such as nuclear spins are the main factors hat disrupt phase coherence of spin qubits. The coherence time can be characterized by the inhomogeneous dephasing time $T_{2}^{*}$ and the homogeneous dephasing time *T*_2_ [25], as illustrated in Fig. 2b. In spin ensembles, $T_{2}^{*}$ predominantly comes from the random static arrangement of spin states and their interaction with the environment. For individual spins, $T_{2}^{*}$ is generally due to the fluctuations in the spin bath state during the averaging process between experiments. In both cases, *T*_2_ represents fast noise processes and is measured using a refocusing π pulse to suppress static and slow fluctuations. For most materials used in quantum applications, the electron spin coherence times $T_{2}^{*}$ and *T*_2_ have orders of microsecond and millisecond respectively, with the potential to be extended to seconds [36], [37], [38], [39]. The limitations in $T_{2}^{*}$ and *T*_2_, mainly attributed to magnetic field fluctuations from the nuclear spin bath, can be mitigated by employing host materials with low spin-full isotope concentrations or improving material quality to reduce high electron spin densities [40], [41], [42]. Cluster correlation expansion calculations and semi-classical models [43], [44], [45] can both be utilized to accurately predict spin coherence times and develop an intuitive understanding of them, enabling further exploration for better host materials. Applying clock transitions [46], dressed states, and dynamical decoupling techniques can help to improve quantum sensing sensitivity and maintain long-term quantum memories.

## 3 Quantum defects in 2D vdW materials

**Fig. 3:**
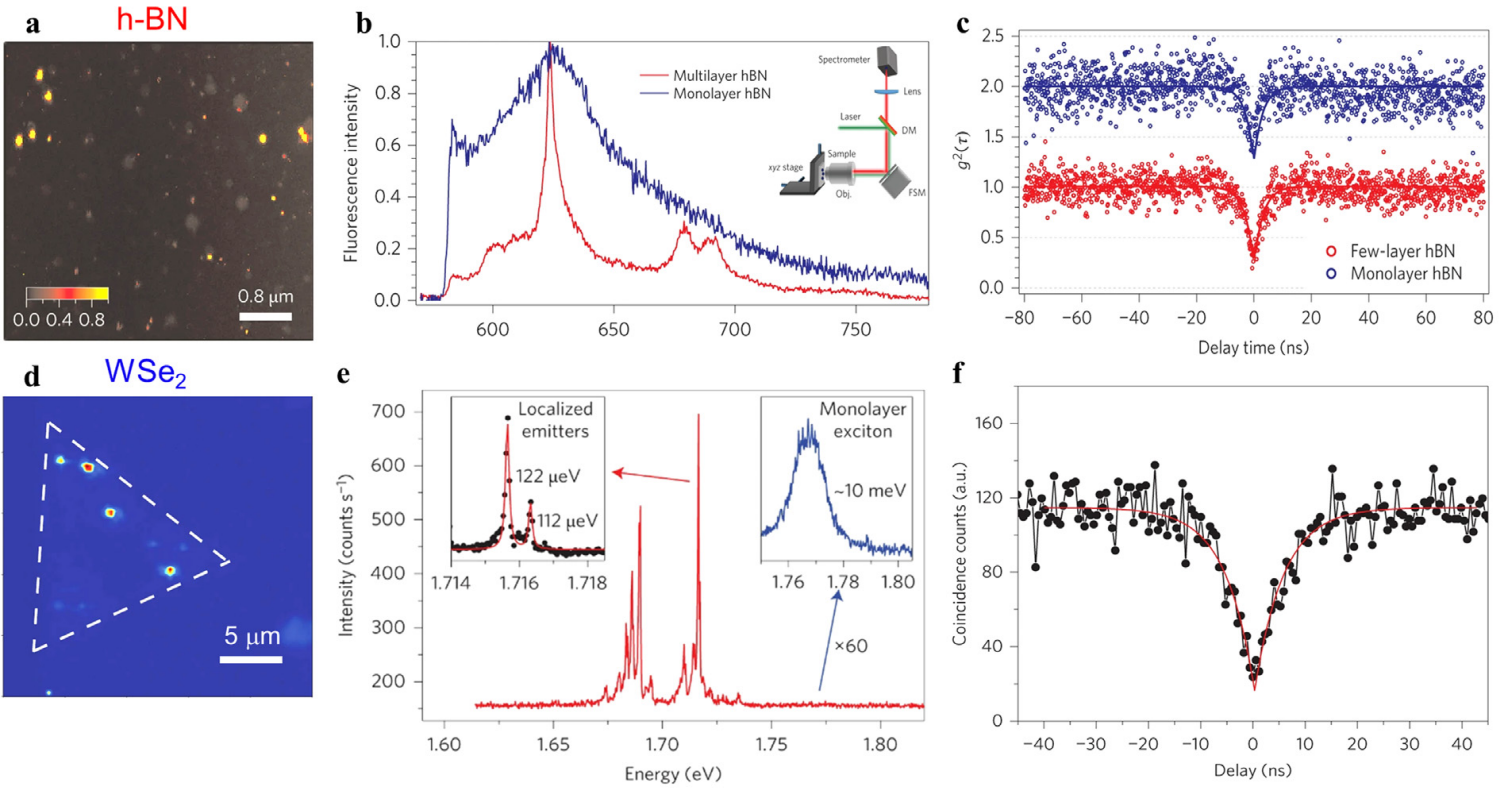
**Optical characterization of SPEs in 2D vdW flakes**. (a-c) The PL intensity map, PL spectra of color centers and antibunching curves from color centers in h-BN at room temperature. (a-c adapted from ref. [53]). (d-f) The PL intensity map, PL spectrum and antibunching curve from color centers in WSe_2_ monolayer. (d-f adapted from ref. [69]).

Thanks to their unique physical properties and potential applications in quantum information science, extensive research has been conducted on 2D vdW materials in recent years [47]. Quantum emitters have been observed in 2D vdW materials including h-BN (Fig. 3a-c) and WSe_2_ (Fig. 3d-f), which has sparked great interest in using these materials for SPEs and potential qubits of quantum computers [18]. The feasibility of creating and positioning quantum defects with the current experimental techniques is a significant advantage of 2D vdW materials for quantum applications, providing new opportunities for scaling-up qubits. Additionally, the properties of these materials, such as vdW interlayer coupling and inert surface without unsaturated dangling bonds, is beneficial to break limitations associated with quantum defects in bulk materials, such as magnetic noise for spin defects and spectral diffusion for color centers.

### 3.1 Quantum defects in h-BN

h-BN, also known as “white graphene,” shares the same hexagonal lattice as graphene, however is composed of alternating boron and nitrogen atoms. The layered bulk h-BN belongs to the space group of P63/mmc, with a lattice constant of 2.504 Å and a layer spacing of 3.30−3.33 Å [48], [49], [50]. Owing to the higher electronegativity of N atom, h-BN exhibits an ionic character, resulting in localized *sp^2^*-hybridized electrons on N atoms and a polarity of B–N bonds. Recent optical spectroscopic studies have shown that bulk h-BN has an indirect band gap of 6.08 eV [51]. However, recent theoretical calculations based on density functional theory (DFT) predicted a direct band gap of 6.47 eV for monolayers [52], while it becomes indirect for few-layer h-BN flakes, although it has not been experimentally confirmed yet.

Interest in h-BN as a promising material hosting quantum defect was sparked by the observation of SPEs at room temperature in 2015 [53], illustrated in Fig. 3a-c. SPEs, as quantum systems that emit single photon during each excitation cycle when optically or electrically activated [2]. It is important to note that photon antibunching is a key characteristic of an SPE, indicated by a dip in the second-order correlation function *g*(*τ*), as shown in Fig. 3c and Fig. 3f. This function be expressed by the formula:

(1) $$g\left(\tau \right)\;=\langle I\left(t\right)I\left(t+\tau \right)\rangle /{\langle I\left(t\right)\rangle }^{2}$$

where *I*(*t*) and *I*(*t + τ*) represent the emission intensity at times t and (*t* + τ) respectively, and 〈 〉 denotes temporal averaging. A perfect single zero-dimensional emitter that is not associated with any background gives *g*(0) = 0. In most case, the experimental value of *g*(0) < 0.5 clearly demonstrates that the emission primarily originates from a single anharmonic emitter or a quantum dot. Usually, *g*(*τ*) can is measured experimentally using a Hanbury–Brown–Twiss (HBT) set-up (Fig. 3c) and can be fitted with the following format:

(2) $$g\left(\tau \right)\;=1-\;A_{1}*\text{exp}\left(-\;\left|\frac{\tau }{t_{1}}\right|\right)\;+A_{2}*\text{exp}\left(-\;\left|\frac{\tau }{t_{2}}\right|\right)$$

where *t*_1_ (*t*_2_) is a characteristic time for photon antibunching (long-timescale bunching), *A*_1_ and *A*_2_ are the antibunching depth and the bunching amplitude, respectively.

Previous experiments have revealed a narrow emission band in h-BN, with a ZPL transition observed in the visible (VIS) and near infrared (NIR) range (1.6 eV–2.2 eV), as shown in Fig. 3b [53,54]. Other experiments have reported luminescence with ZPLs in the ultraviolet (UV) range (4.1 eV–5.3 eV) [55,56]. These results suggest that the observed emitters in h-BN may be attributed to various electronic states caused by factors, such as the variations of structural composition, the uncertainty of defect charge state, and the change of local strain and dielectric environment. Subsequent studies have focused on producing high-quality 2D h-BN with well-controlled point defects through techniques, such as heating, etching, and irradiation with high-energy particles. Furthermore, the defect states in h-BN can be modulated by external stimuli such as electrical field, magnetic field, and strain field. Notably, defect engineering enables control of functional properties of h-BN in the atomic scale.

**Fig. 4:**
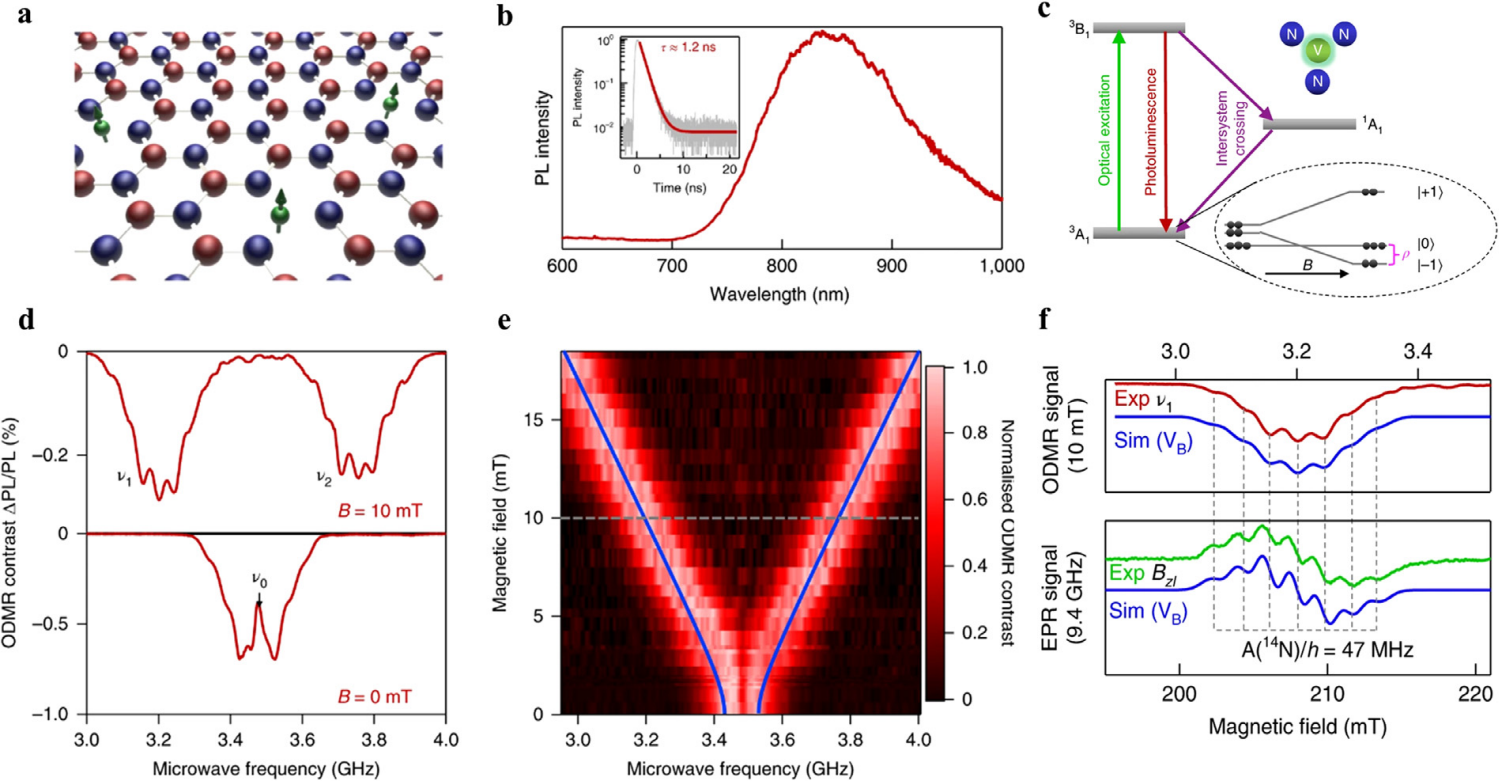
**ODMR of an h-BN single crystal at room temperature**[23]. (a) Crystal structure of an h-BN monolayer with alternating boron (red) and nitrogen (blue) atoms. The spins of $V_{B}^{-}$ is labeled by green arrows. (b) PL spectrum of defect $V_{B}^{-}$. Inset image: the excited-state lifetime is fitted with an exponential function. (c) ODMR spectra of h-BN flake as a function of frequency at B = 0 mT (bottom) and B = 10 mT (top). (d) Dependence of ODMR frequencies on the magnetic field (B || c). (e) Simplified energy-level scheme of optical transitions among triplet excitation state and ground state. (f) Hyperfine splitting of ODMR *v_1_* (red trace) line and of EPR *B_Zl_* (green trace) transition.

On the other hand, advanced first-principles calculations were used to evaluate different defects in single-layer h-BN for their thermodynamic charge transition levels. The study revealed that the center $V_{N}C_{B}$ (nitrogen vacancy adjacent to a carbon substitution of boron) can produce a spin triplet (*S* = 1) ground state [57], indicating the possibility of h-BN hosting defect qubits. Additionally, Later, Abdi et al. theoretically predicted that the negatively charged boron vacancy ($V_{B}^{-}$) in h-BN is the most suitable quantum defect for scalable qubit systems and SPEs [58]. This prediction was experimentally supported by optically detected magnetic resonance (ODMR) and EPR measurements in 2020 [23], as shown in Fig.4. The $V_{B}^{-}$ center in h-BN has $D_{3h}$ point-group symmetry and exhibits strong photoluminescence (PL) emission at $\lambda_{max}=800\;\text{nm}$ at room-temperature (Fig.4b). Its electronic structure is similar to that of diamond *NV*^−^ center, with a ground state of a spin triplet state (^3^$A_{2}$) and a ZFS of ∼3.47 GHz between spin sublevels *m_s_* = 0 and *m_s_* = 1, as shown in Fig.4c. The ODMR spectrum in Fig.4d shows that $V_{B}^{-}$ center exhibit two distinct resonances, $\nu_{1}$ and $\nu_{2}$, symmetrically located around the frequency $\nu_{0}$. These resonances correspond to the $\Delta m_{S}=\pm 1$ spin transitions between the triplet energy sublevels with a completely lifted threefold degeneracy. The splitting is a result of the dipolar interaction between the unpaired electron spins. Neglecting hyperfine coupling, the spin state of a given defect can be represented by a spin Hamiltonian with *Z* as the principal symmetry axis, being parallel to the c-axis of the h-BN crystal [23]:

(3) $$H=D\left(S_{z}^{2}-S\left(S+1\right)/3\right)+E\left(S_{x}^{2}-S_{y}^{2}\right)+g\mu_{B}BS$$

where *D* and *E* are the ZFS parameters, *S* is the total electron spin, *g* is the Landé factor, $\mu_{B}$ is the Bohr magneton, *B* is the static magnetic field and *S_x,y,z_* are the spin operators. The evolution of the ODMR spectrum of the $V_{B}^{-}$ center with a magnetic field *B* parallel to the hexagonal *c* axis (B∥c) of h-BN can be described by the following equation:

(4) $$\nu_{1,2}\;=\;\nu_{0}\pm \;\frac{1}{h}\;\sqrt{E^{2}+{\left(g\;\mu_{B}B\right)}^{2}\;}$$

with $\nu_{0}=D/h=3.48\;\text{GHz}$ and a small off-axial component of the ZFS E/h=50MHz, demonstrating a highly symmetrical, almost uniaxial, defect structure.

Furthermore, pulsed spin resonance measurements of $V_{B}^{-}$ centers in h-BN showed a reasonably long spin relaxation time $T_{1}\approx 18\;\mu s$ at room temperature. At cryogenic temperature, this relaxation can be substantially increased by three orders of magnitude. The spin–spin coherence time *T*_2_ was found to be 2μs at 300 K [59]. The spin relaxation of $V_{B}^{-}$ centers is determined by spin-phonon interaction, following a power law of $T^{-5/2}$. The maximum spin-relaxation time of $V_{B}^{-}$ was determined to be $T_{1}\approx \;25\;\mu s\;$and $T_{2}\approx \;7.5\;\mu s$ at room temperature, by suppressing the inhomogeneous broadening resulted from the surrounding nuclear bath with a spin hole-burning technique. Additionally, Liu et al. demonstrated that the $V_{B}^{-}$ spin ensemble in h-BN can be coherently manipulated with Rabi oscillation at room temperature [60]. Moreover, they also detected *T*_1_ and performed the spin-echo and Ramsey-interference experiments. It was found that *T*_1_ was almost independent on the magnetic field, while the experimental results of the Rabi oscillation, spin echo, and Ramsey oscillation differed significantly when magnetic fields were either weak or strong. Especially, $V_{B}^{-}$ centers exhibit a strong electron nuclear spin coupling, as observed in the magnetic-field-dependent Rabi oscillations. The ^14^N nuclear spins can be tuned by microwave and polarized at strong magnetic field. The ^11^B nuclear spin bath in h-BN s plays a significant role in the dynamics of $V_{B}^{-}$ spin relaxation. By using a magnetic field, the relaxation of spin can be prevented, and the oscillation of nuclear-spin caused by microwave can be increased, consequently affecting $V_{B}^{-}$ spin dynamics.

However, investigating spin defects in h-BN crystals with the natural boron isotopes poses a difficult task in revealing the microscopic structure and hyperfine spectra of optically active spin defects [61]. This is due to the complexity of these crystals, which consist of vacancies, anti-site defects, and extrinsic substitutional or interstitial impurities. To circumvent this problem, Haykal et al. utilized h-BN crystals with only one boron isotope to explore the spin coherence features of $V_{B}^{-}$ centers [62]. A slight rise in the spin coherence time *T*_2_ in ^10^B-enriched samples is observed. Cross-relaxation spectroscopy further was used to determine that dark electron spin impurities may contribute to the spin decoherence of the $V_{B}^{-}$ center in h-BN. In addition, h-BN with well-isolated single defects were prepared and investigated recently by Stern et al. [63]. The ODMR contrast of a single-defect was found to exceed 30%, which is approximately 100 times greater than 0.4% of the high-density ensemble.

**Fig. 5:**
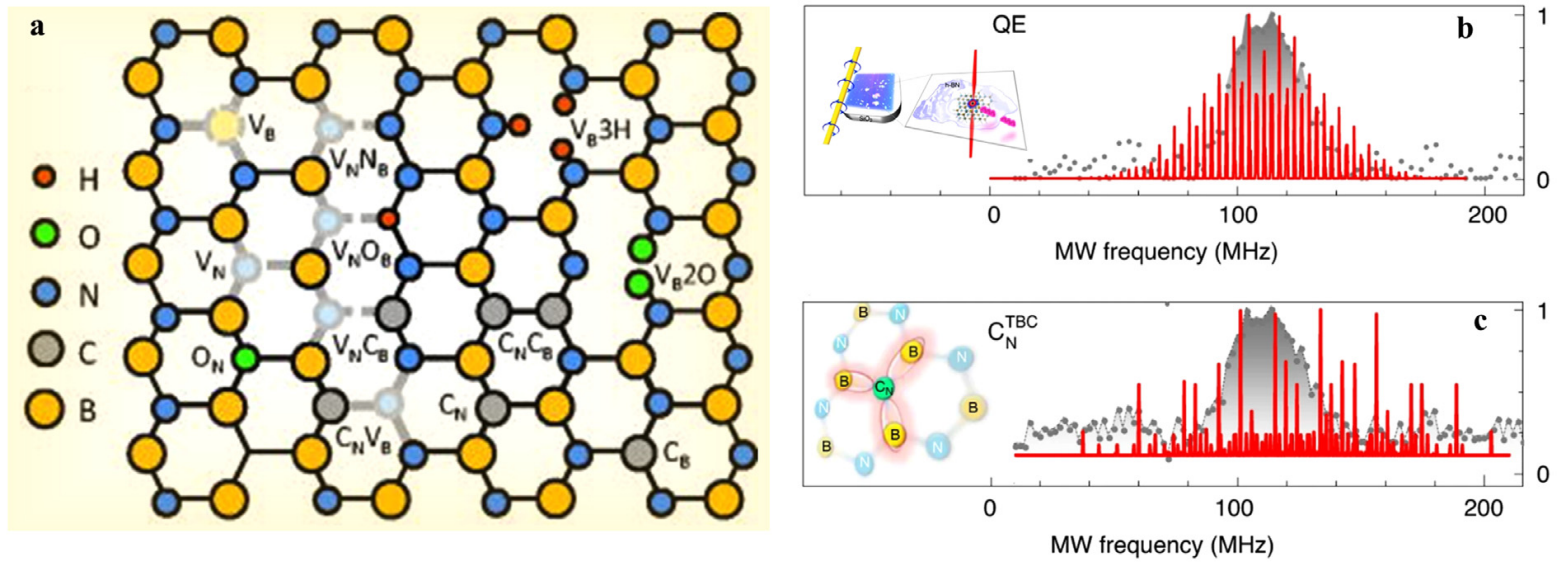
**Atomic defects in h-BN**. (a) Structural characteristics of various types of point defects as the quantum emitters in h-BN [16]. (b-c) Simulation (red) and experiment (grey) of ODMR spectra from the defect $C_{N}^{TBC}$[64].

In addition to $V_{B}^{-}$, the lattice structure of h-BN also contains various intrinsic defects, antisite defects, and unintentional impurities. As shown in Fig. 5a, plausible defect structures include nitrogen vacancy $V_{N}$, carbon substitutional $C_{B}$ and $C_{N}$, oxygen substitutional $O_{N}$, silicon substitutional $Si_{B}$, a vacancy next to a substitutional atom such as nitrogen $V_{N}N_{B}$ and $V_{N}O_{B}$, and a double substitutional carbon defect $C_{N}C_{B}$ [11], as shown in Fig. 5a. Recent experiments have captured ODMR signals (Fig. 5b) from individual or a few defects at room temperature for color centers in h-BN, which were assumed to be $C_{N}$ defects after comparing the detected hyperfine signatures with the calculated ones (Fig. 5c) [64].

### 3.2 Quantum defects in TMDs

TMDs are typical semiconductors with an energy gap in the NIR to VIS range, making them suitable for opto-electric devices such as photodetectors, solar cells and biosensors [19,^65^,66]. Crystalline 2H-TMDs belong to the space group of P63/mmc and are composed of stacked *X* − *M* − *X* layers, with a plane of metal atoms (*M* = Mo, W) sandwiched between two trigonal planes of chalcogen atoms (*X* = *S*, Se, Te). While single sheets are coupled through weak vdW interaction, atoms within the *X* − *M* − *X* layer are bonded covalently. These characteristics ensure that layered TMDs can be easily thinned into single and a few layers through mechanical and chemical peeling, thereby modulating physical properties. In addition, monolayers of TMDs such as WS_2_ and MoS_2_ possess a direct bandgap at K point in the Brillouin zone, whereas multilayers of these materials have an indirect bandgap. Furthermore, due to strong spin-orbit coupling, monolayer TMDs exhibit significant spin splitting in the valence and conduction bands, which are on the order of several hundreds and tens of millielectronvolts, respectively. Finally, monolayer TMDs with time-reversal symmetry and broken inversion symmetry gives rise to inequivalent valleys with opposite pseudospin (valley degree of freedom) in momentum space, denoted as |K〉 and $|K^{\prime }\rangle$. Therefore, monolayer TMDs possess many peculiar physical properties, such as the chirality-dependent optical selection rules, the spin-valley locking effect, and the valley Hall effect, laying a reliable physical foundation for developing quantum computing with valley pseudospin qubit of 2D vdW materials [67].

Unlike h-BN, research on optical addressing spin defects in TMDs is still in its early stages, mainly focusing on theoretical predictions. However, due to advances of SPEs in TMDs, exploring the possibility of defect spin qubits in TMDs has great appeal. The discovery of SPEs in TMDs was attributed to four representative works reported concurrently in 2015 [68], [69], [70], [71]. Specifically, PL emissions with energies lower than the intrinsic excitonic emission of 20–100 meV were observed from monolayer WSe_2_ at cryogenic temperature. These SPEs are characterized by photon antibunching, narrow linewidths down to 0.1 meV, linear polarization and lifetimes on the order of 1 ns. In addition, the neutral localized excitons of TMDs typically contain a fine-structure split doublet, featuring orthogonal linear polarization and a splitting of 0.4–0.8 meV. This splitting is usually attributed to either to electron–hole exchange interaction or the strong Coulomb interactions, depending on the magnitude. Subsequent studies also found SPEs in other TMDs, such as MoS_2_, WS_2_, and MoSe_2_ [72], [73], [74]. The structural origins of SPEs in these TMDs are subject to debate and mainly can be classified into two types. The first type is a color center with electronic states located deep in the bandgap, exhibiting the characteristics of an isolated artificial atom. The local strain characteristics are usually at the scale of 100 nm, which is significantly larger than the exciton Bohr radius of TMDs. Although local wrinkles at the stressor sites could make excitons localized at the 10-nm scale [75], the origin for the SPE could not be solely stem from local strain, but rather from the interplay or combination of local strain and disorder or defects. The second type is called a quantum dot (QD). The emission is due to bound excitons restrained by a potential field resulting from local strain. Recently, SPE up to 150 K was observed in Wse_2_ by combining controlled strain and electron-beam-induced structural defects, characterized by a relatively high yield and biexciton formation [76]. Furthermore, SPEs were successfully fabricated via encapsulation of MoS_2_ monolayers between two h-BN layers after irradiation, exhibiting long excited state lifetimes and clear antibunching [77]. The h-BN layers act as a protective barrier, shielding the 2D material from surrounding ambient gases as well as the substrate, and reducing the effect of doping, surface roughness, and any residues caused by nanofabrication.

**Fig. 6:**
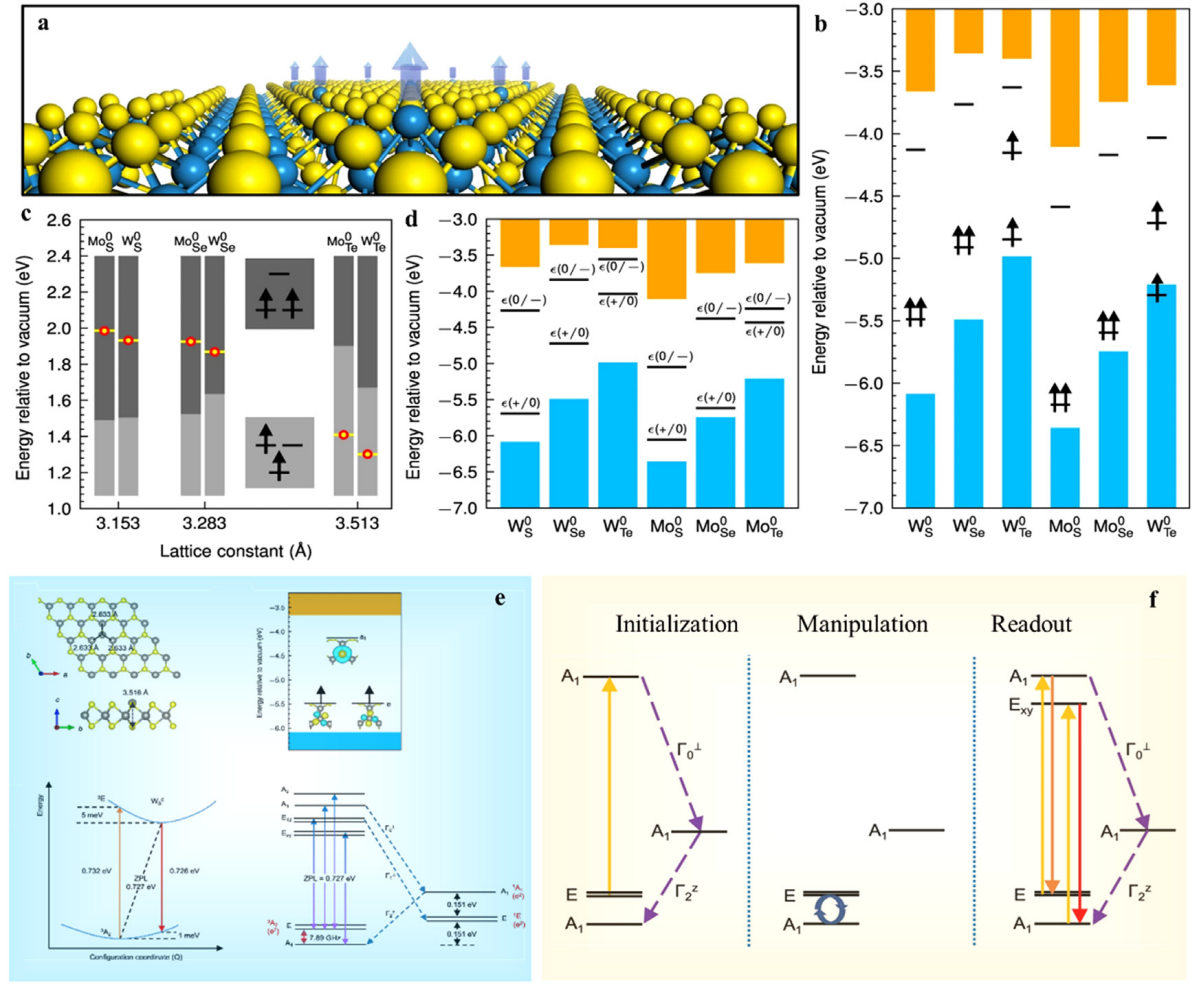
**Anion-antisite defect qubits in six 1H-TMDs**[78]. (a) Geometry of the $M_{x}^{0}$ antisite point defect in single-layer 1H-TMD. (b) Defect levels of $M_{x}^{0}$ in band gaps of 1H-TMDs with ground-state triplets. (c) The correlation between the level splitting of defect and the relative z-positions of the anion antisites and neighboring cations. (d) Thermodynamic transition energy levels of antisite point defects in six 1H-TMDs. (e) Geometric and electronic properties for the neutral antisite defect $W_{s}^{0}$ in WS_2_. (f) An operational loop based on the antisite qubit $W_{s}^{0}$.

For spin defects, the advanced theoretical investigation using spin Hamiltonians and a cluster expansion method showed that the spin coherence time *T*_2_ of an isotopically purified layer of MoS_2_ can be long as the order of 30 ms [24], indicating the promising potential of TMDs as suitable host materials for multiple-qubit operation. After that, Tsai et al. proposed a hypothesis that incorporated symmetry constraints and the host electronic structures to identify potential candidates of defect-spin qubits with stable triplet ground states. Through a high-throughput search they discovered thermodynamically stable neutral anion-anti-site defects in six monolayer 1H-TMD compounds [78]. The Fermi level in the gap with the most stable neutral charge state has energy windows at 1.43 eV, 0.88 eV, 0.48 eV, 1.01 eV, 1.24 eV, and 0.19 eV for $W_{S}^{0}$, $W_{Se}^{0}$, $W_{Te}^{0}$, $Mo_{S}^{0}$, $Mo_{Se}^{0}$, and $Mo_{Te}^{0}$, respectively, as depicted in Fig. 6. By comparing the position of defect levels in the bandgap of these materials, it is concluded that $W_{S}^{0}$ and $W_{Se}^{0}$ are the most viable defect qubits in 2D TMDs. The calculated electronic structure of $W_{S}^{0}$ hosts the triplet ground state ^3^$A_{2}$ and the triplet excited state ^3^*E*, which are identical to the defect levels of *NV* center in diamond. The ZPL for the internal transition from the triplet ground to the excited state is 0.727 eV that is equivalent to a wavelength of approximately 1.7 µm in the NIR range. The positions of singlet states ^1^*E* and ^1^$A_{1}$ are essential for studying nonradiative decay paths that bridge the triplet and singlet states, and they are estimated by considering the Coulomb interaction. As shown in Fig. 6e, the energy difference between ^1^*E* and ^3^$A_{2}$ of $W_{S}^{0}$ is 0.151 eV, and the computed ZFS of 7.89 GHz is more than double the 2.88 GHz found in diamond for $N_{V}$ center. Three allowed intersystem-crossing paths $\Gamma_{0}^{⊥}$, $\Gamma_{1}^{⊥}$, and $\Gamma_{2}^{z}$ are determined by analyzing the spin quantum numbers and IRs of tensor products of wavefunctions and spinors. As shown Fig. 6f, initializing, manipulating, and reading out the TMD-based anti-site qubit (Fig. 6f) are similar to the operations performed on defect qubits in the $N_{V}$ center of diamond.

**Fig. 7:**
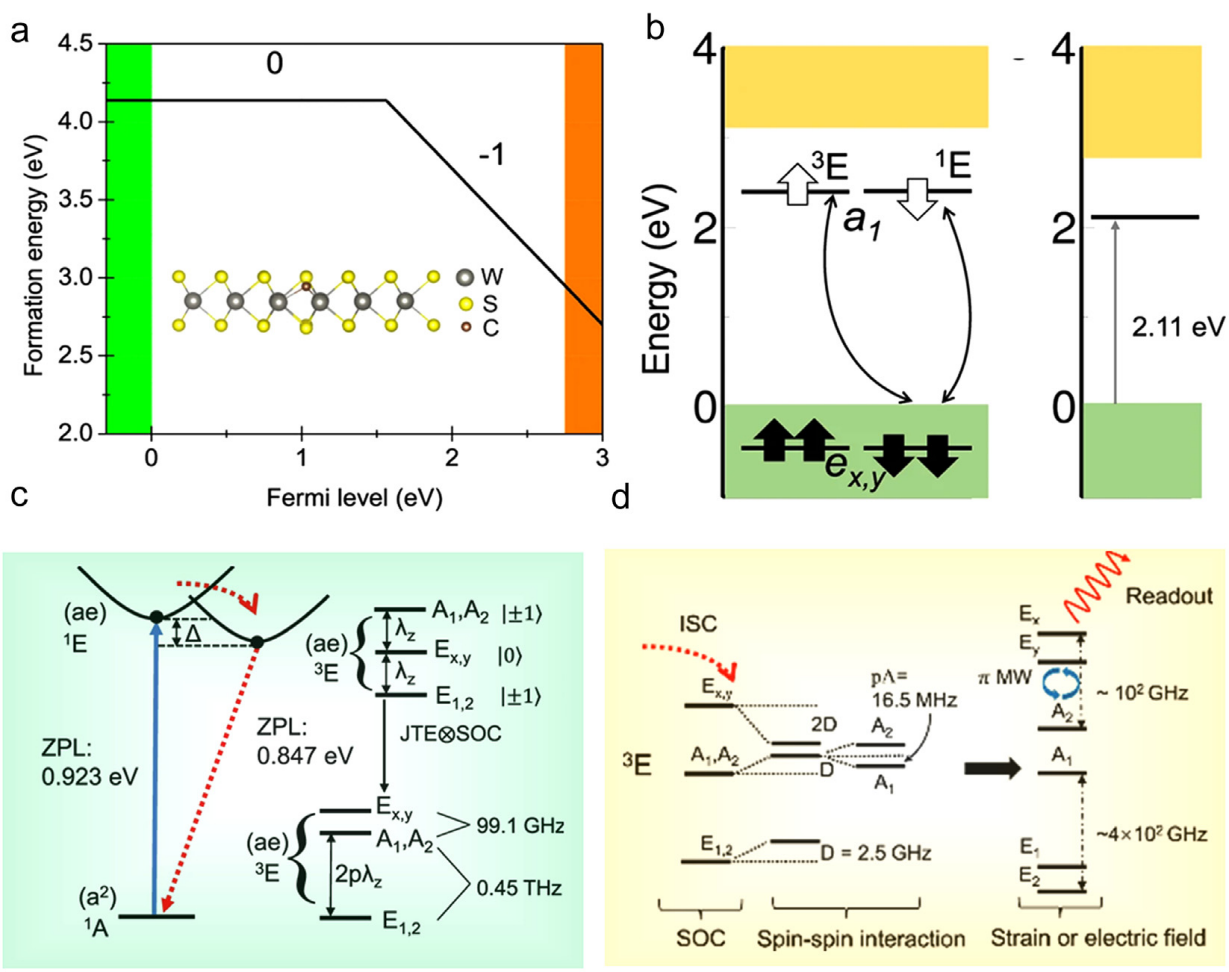
**Carbon defect qubit in WS_2_**[79]. (a) Formation energy as a function of Fermi-level for *C_S_* defect. The energy diagram without SOC effect (b) and with SOC effect (c) for neutral *C_S_* defect at ground state. (d) The possible optical transition among ground state and excited states of $C_{s}^{0}$.

Another candidate of spin qubits is the carbon defect in WS_2_, specifically a carbon atom substituting Sulphur ($C_{s}$) atom as shown in Fig. 7, which can be precisely and on demand modulated at atomic level with scanning tunnelling microscopy (STM) tips [79]. Through DFT HSE calculation, Li et al. determined the electronic structure and optical properties of $C_{s}$, revealing its potential as a qubit candidate. The spin can be initialized and read out optically, and a coherent state can be prepared through microwave excitation. As shown in Fig. 7c, the ground state is a closed shell singlet ^1^$A_{1}$. A spin-conserving optical transition exists between the ground state ^1^$A_{1}$ and an excited-state ^1^*E* with ZPL of 0.923 eV (about 1340 nm). Upon reaching ^1^*E*, a highly selective intersystem crossing (ISC) occurs to the $m_{s}\;=\;0\;$of $E_{x,y}$ state of triplet ^3^*E*, and its fine structure is determined by the intricate interaction between phonons, orbitals and spins. The ISC rate from ^1^*E* can be estimated with the equation:

(5) $$\tau_{ISC}\;=\left(\frac{2\pi }{h}\right)\lambda_{z}^{2}F\left(\Delta E\right)$$

where *F* is the function representing overlap between the vibrational spectra of singlet and triplet, and *ΔE* is the energy splitting between them. The ISC lifetime was estimated in the order of picosecond that is much shorter than the radiative lifetime of the ^1^*E* state. Moreover, optical pumping can be used to initialize the $E_{x,y}$ state as one of the quantum levels. The optical lifetime of *^3^E* is calculated to be 145 ns, with a radiative rate of 1.10 (2π) MHz in angular frequency. As shown in Fig. 7d, the electron spin qubit state can be optically initialized and read out. Recent studies [80] has shown, however, that the coherence time of the *S* = 1 qubit in WS_2_ should be roughly 11 ms, being caused by the large hyperfine interaction between the electron and nuclear spins in the vicinity of the defect. In addition, ^13^*C* has a large hyperfine interaction, thus it should be avoided as an ancilla qubit, as this would cause decoherence.

## 4 Formation of defects in 2D vdW materials

The formation of defects in 2D vdW materials can be achieved through two approaches: the top-down and bottom-up methods. The former includes mechanical exfoliation and irradiation, while the latter includes molecular beam epitaxy (MBE) and chemical vapor deposition (CVD). It is worth noting that some among these methods are compatible with the modern micro/nanofabrication, including focused ion beam (FIB), electron beam lithography (EBL) and direct laser writing (DLW). These techniques provide the good opportunities for building multiple qubits and quantum networks.

### 4.1 Direct synthesis for defects

The typical strategy for forming defects in 2D vdW materials is to adjust the synthesis parameters for achieving stoichiometry changes during material growth. These parameters include growth temperature, substrates, precursor concentration and pressure. In the previous studies, CVD has shown promise as a growth method, due to its flexibility, controllability, and potential for mass production. By adjusting synthesis parameters, the desired defects and other expected properties can be selectively obtained. For example, substrates are critical for the CVD synthesis of defective films, due to the existence of physicochemical states. For the mostly used SiO_2_/Si substrate, it is usually to form intrinsic defects such as vacancies and pits during the growth process, caused by dangling bonds or defects on the one end. this can make its surface chemically active and rough, resulting in an aperiodic lattice arrangement during the atomic deposition. In addition, the SPEs of h-BN grown on normal Cu and pre-oxidized Cu mostly exhibits the emission in the range of 600–650 nm, whereas for as-grown h-BN on Cu/Ni, the emission wavelength of the SPEs shifts to 550–600 nm, due to the different types of defects [81].

It is well known that carbon-based defects occupy deep level states within the h-BN bandgap, which typically leads to deep UV emissions depending on material purity [55,56]. In the previous study on the synthesis of bulk h-BN crystals, Kubota et al. presented that the near band edge UV emission at 215 nm from h-BN is suppressed by the amount of carbon and oxygen incorporated in the material, whose impurity concentrations have to be below $1\times 10^{18}/cm^{3}$ to maximize these emissions [82]. Recently, Bourrellier et al. identified the quantum nature of the mid-UV emissions of h-BN, where SPEs are contributed by carbon-based point defects in the h-BN crystal [83]. Indeed, the utilization of carbon-rich metal organic sources make it difficult to prevent the incorporation of carbon in h-BN films. Through comparing samples grown by MOVPE, MBE and HOPG conversion, Mendelson et al. identified a direct correlation between the formation of SPEs and the introduction of carbon as a precursor/substance [84]. And defect ensembles were confirmed to display room-temperature ODMR.

Moreover, the previous theoretical study of DFT indicated that formation energy of S vacancy ($V_{S}$) is relatively lower in MoS_2_, especially in the Mo-rich conditions, so $V_{S}$ is the richest defect in practical growth conditions [85]. An et al. achieved hexagonal WS_2_ with heterogeneous defects (both $V_{S}$ and W vacancy ($V_{W}$)) based on the accumulation model for the remnant W-precursors [86]. Zhou et al. demonstrated that $V_{S}$ and sulfur divacancies ($V_{S2}$) are able to be introduced into the MoS_2_ flakes while keeping the crystalline feature of the entire MoS_2_, by tuning the hydrogen flow rate in the H_2_/Ar mixed carrier gas [87]. In addition, it was discovered that the primary type of point defects in monolayer MoS_2_ is highly dependent on the sample synthesis methods [88]. It is observed that anti-site $Mo_{S}$ defects are the primary point defects in physical vapor deposited (PVD) MoS_2_, while $V_{S}$ defects are the most typical defects in monolayer MoS_2_ grown by mechanical exfoliation and CVD. Jeong et al. found that metal vacancies play a prevailing role in determining macroscopic material characteristics of single-crystalline 2H-WS_2_ monolayers grown by CVD [89]. The hexagonal shape of WS_2_ flakes is composed of alternating triangular $V_{S}$-rich and $V_{W}$-rich domains without forming clear defective grain boundaries. The $V_{W}$-rich domain with deep-trap states presented an electron-doping effect, resulting in a decrease of electron mobility and PL emission by one order of magnitude compared to the $V_{S}$-rich domain with shallow donor states.

### 4.2 Irradiation engineering for defects

Irradiation induced defects with high-energy particles and photons had been extensively studied in bulk semiconductors such as Si and GaN before, which come from the ionization, electronic excitation, and nuclear displacement damage. While energetic particles give rise to defects in both bulk and 2D vdW materials [90], the recent studies have shown that the response of 2D vdW materials to irradiation may differ from that of bulk systems. This is due to backscattered ions and sputtered atoms from their supporting substrates. As schematically illustrated in Fig. 7a, the number of defects per ion impact initially increases with ion energy in both 2D and bulk materials, but it starts to decrease at some stage in 2D materials due to the drop in the cross-section for atom displacement and the absence of collisional cascades.

**Fig. 8:**
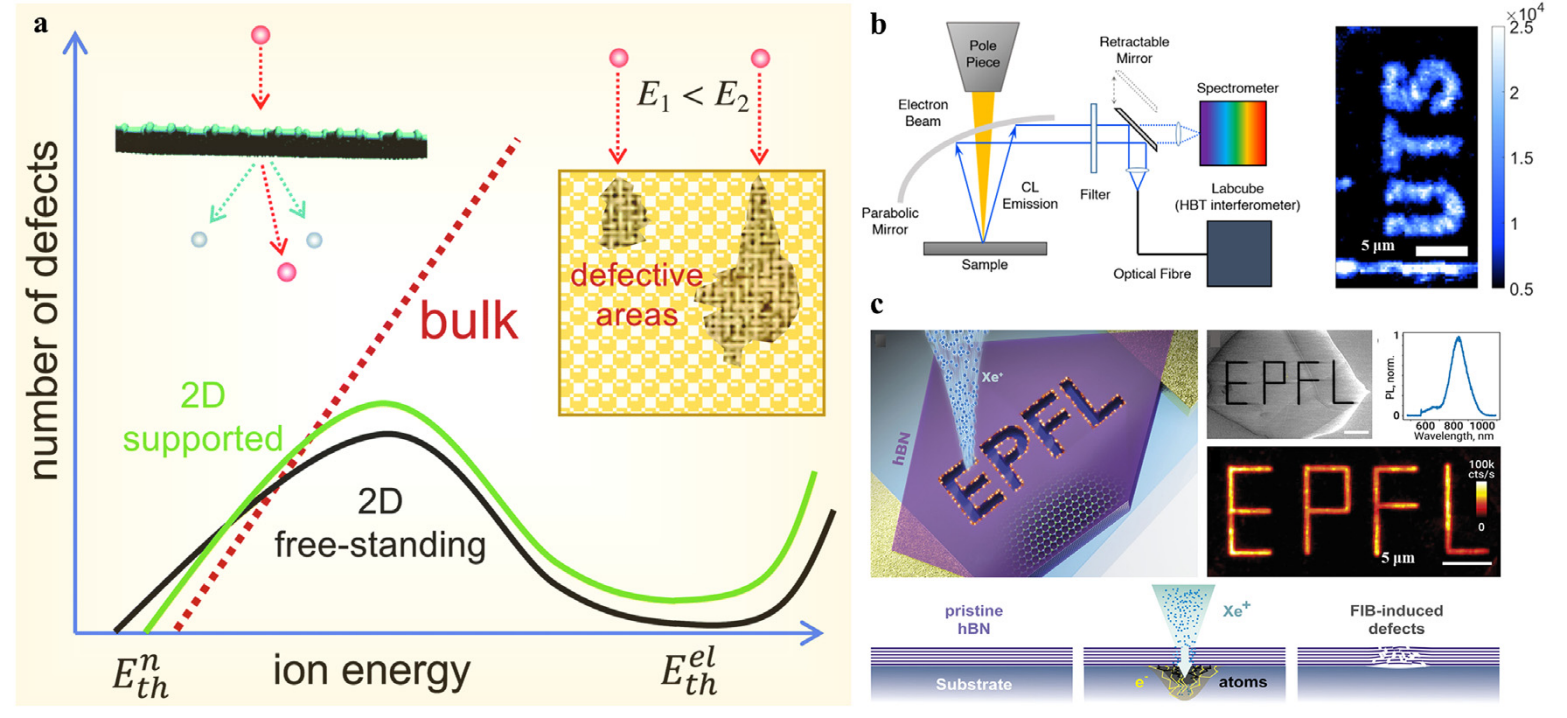
**Irradiation engineering for defects.** (a) Schematic representation of defect fabrication in bulk and 2D materials under ions irradiation [90]. (b) Blue emitters in h-BN based on electron beam site fabrication [91]. Left: The electron beam irradiation setup. Right: Confocal PL image of “UTS” in h-BN engineered by electron beam fabrication. (c) Production of optically active defects in exfoliated h-BN flakes using FIB fabrication [92]. Upper left: the defect in h-BN flake writing process with Xe FIB. Upper right: SEM and Fluorescence image of a FIB-induced patterned flake. Bottom: Simplified defect generation process using FIB.

In previous studies, electron and ion irradiations have been commonly used to generate defects in 2D vdW materials. These methods are convenient and widely applied in nanoscience research, allowing for accurate and spatially selective defect sites by standard nano-lithographic techniques such as EBL (Fig. 8b) and FIB (Fig. 8c). For example, Gale et al. developed the direct-writing electron beam technique for blue emitters in h-BN based on a dual beam FIB/SEM microscope [91]. Importantly, no additional annealing was needed after electron irradiation. In addition, it was demonstrated that individual quantum emitter can be created at the specific site in h-BN by tuning the spot size and dose time of an electron beam of 15 keV. Similarly, Glushkov et al. fabricated an array of optically active spin defects on pristine h-BN flakes by using a commercial FIB system with Xe ion irradiation [92]. Kianinia et al. demonstrated the creation of optically addressable spin defects based on the $V_{B}^{-}$ center in exfoliated hexagonal boron nitride using various focused ion beams such as nitrogen, xenon, and argon [93]. Lithographic techniques offer new opportunities for engineering defect formation with well-ordered positions and tunable sizes.

Plasma irradiation is another common method to induce defects into 2D vdW materials. It is widely used for surface cleaning, functionalization, passivation, and etching materials. Compared to ion irradiation, plasma processing involves more chemical reactions of species on the surface of samples. In short, the reactive gas is ionized into plasma by radio power, and different energy-activated chemical groups absorb this energy. High-energy ion beams then bombard the target materials, breaking their chemical bonds and producing surface atomic defects. By adjusting the gas source, output power, and treatment duration, the type and density of defects can be controlled, according to the formation energy of different defects. For example, oxygen plasma treatment introduces O–Mo bonds in MoS_2_ flakes, while argon plasma can be used for the creation of sulfur vacancies [94,95]. In addition, Fischer et al. used plasma irradiation plasma irradiation followed with annealing to successfully fabricate two types of luminescent centers. Their PL line shapes match well with $V_{N}C_{B}$ and $V_{B}^{-}$ luminesce respectively, calculated by using molecular dynamics simulations [96].

**Fig. 9:**
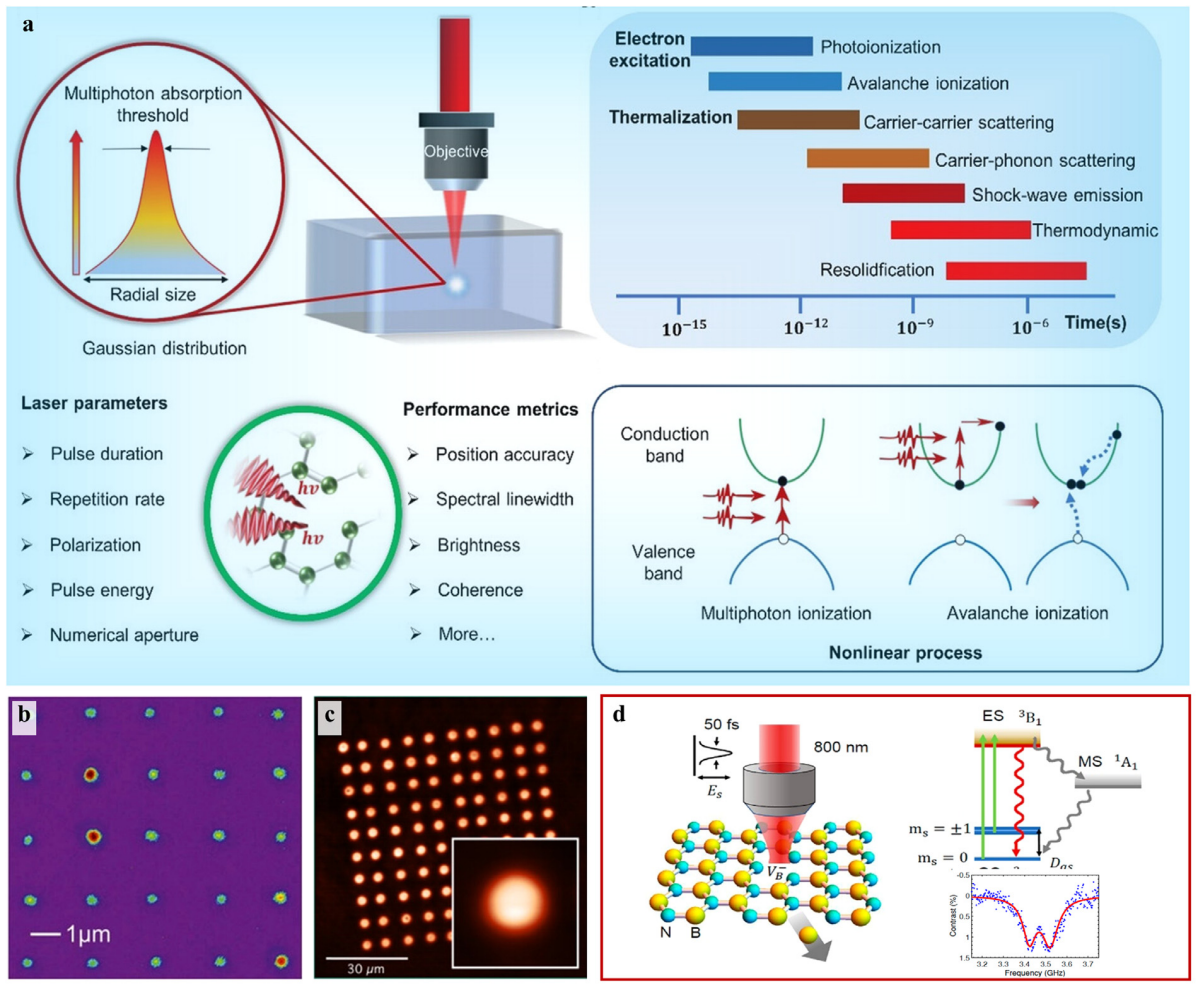
**The schematic illustration of DLW technique for color centers.** (a) Overview of DLW: focused laser field strength presenting a Gaussian distribution, significant laser parameters, the ultrafast time scale of the physical phenomena for laser-matter interaction and nonlinear absorption process [97]. (b) Fluorescence map of a high-yield 5 × 5 array with single *NV* centers by DLW [98]. (c) PL image of 10 × 10 array of *NV* centers by DLW with a magnified image of individual pixel [99]. (d) Schematic of energy-level structure and optical transitions of $V_{B}^{-}$ and the ODMR curves of the $V_{B}^{-}$ centers by DLW [101].

The direct laser writing (DLW) has recently emerged as a powerful way to create color centers in solid-state materials, which is a method of depositing energy at optical focus using a laser pulse [97]. The DLW approach can deliver energy inside of solids and scan it three-dimensionally, thanks to the nonlinear optical process. Unlike high-voltage electron beam irradiation, DLW can be performed at ambient environment, offering simplicity and flexibility. The laser does not induce lattice damage along the optical path, as ions or electrons do. Patterns fabricated with DLW depend on various factors such as the energy of pulse, its duration, its repetition rate, the polarization, and the numerical aperture of the lens (Fig. 9a). The electrons can be excited through multiple-photon absorption, such as avalanche ionization and multiphoton ionization. The nonlinear absorption in the DLW process by a femtosecond laser occurs much faster than lattice relaxation. DLW offers a promising approach for enabling site-specific control of *NV* centers in diamond. The highly nonlinear interaction between the laser pulses and the diamond lattice allows for precise positioning *NV* centers with accuracy far exceeding the diffraction limit [98], as shown in Fig. 9b. DLW further was utilized to create the *NV* center array (Fig. 9c) in the regime of nano-ablation on the diamond surface with a positioning precision down to hundreds of nanometers with 266 nm femtosecond pulse lasers [99]. The correlation between the *NV* concentration and the irradiation parameters may be attributed to the process of vacancy formation on the surface of diamond, as well as the laser-stimulated diffusion of these vacancies in the bulk. Especially, the DLW provides a potential route to sever bonds and form multiple kinds of defects in h-BN. It has been reported that the DLW can generate SPEs in h-BN monolayers and flakes at the desired position, and the emission properties of the SPEs are consistent with the anti-site defects $N_{B}V_{N}$ [100]. As shown in Fig. 9d, the optically active spin defects $V_{B}^{-}$ were controllably created for spin-based quantum applications by femtosecond laser writing, which presents good contrast in ODMR measurement [101]. It is suggested that laser fabrication will open up more possibilities for 3D high-density data storage using color centers, as technology and ideas continue to develop.

There are also other high-energic particles used to induce defect in 2D vdW materials, such proton, neutron and γ-rays. Tedeschi et al. demonstrated that proton irradiation has a major impact on the surface morphology of TMD crystals [102], but protons will be virtually transparent to other 2D crystals such as graphene and h-BN. Protons are able to penetrate the top layer of TMDs, leading to the generation and gradual increase of molecular hydrogen in the first interlayer area. Blistering of one-monolayer thick domes found on the crystal surface, causes the dark bulk material to become an effective light emitter. Moreover, the previous research by Toledo et al. suggested that neutron irradiation creates a different kind of paramagnetic defect with visible optical absorption [103]. Xiong demonstrated that the PL properties of MoS_2_ flakes can be greatly improved, when the irradiation fluence of neutron accumulates to $3.2\;\times \;10^{9}/cm^{2}$ [104].

Compare all the above methods of producing defects in 2D vdW materials, the irradiation method with energetic particles presents the most promising for future quantum applications. This method possesses great advantages on engineering the formation process of defects with well-ordered positions and tunable sizes. In particular, some among these methods are compatible with the modern micro/nanofabrication, providing new opportunities for achieving controllable generation of qubits and manufacturing multiqubit quantum systems and networks.

## 5 Modulation of defects in 2D vdW materials

While quantum defects such as $V_{B}^{-}$ in h-BN exhibits promising application potential in quantum information, especially in quantum sensing and imaging, a critical limitation of these quantum defects is their low brightness originated from spectral diffusion that manifests itself as a high intrinsic non-radiative decay rate, which leads to a rather low ODMR contrast. To address the problem of low quantum yield, quantum emitters can be modulated by external methods such as electrostatic gating, optical stark effect, integrated plasmonic cavities, therefore activating certain intrinsic defects as well as the emission rates.

### 5.1 Electrical modulation

**Fig. 10:**
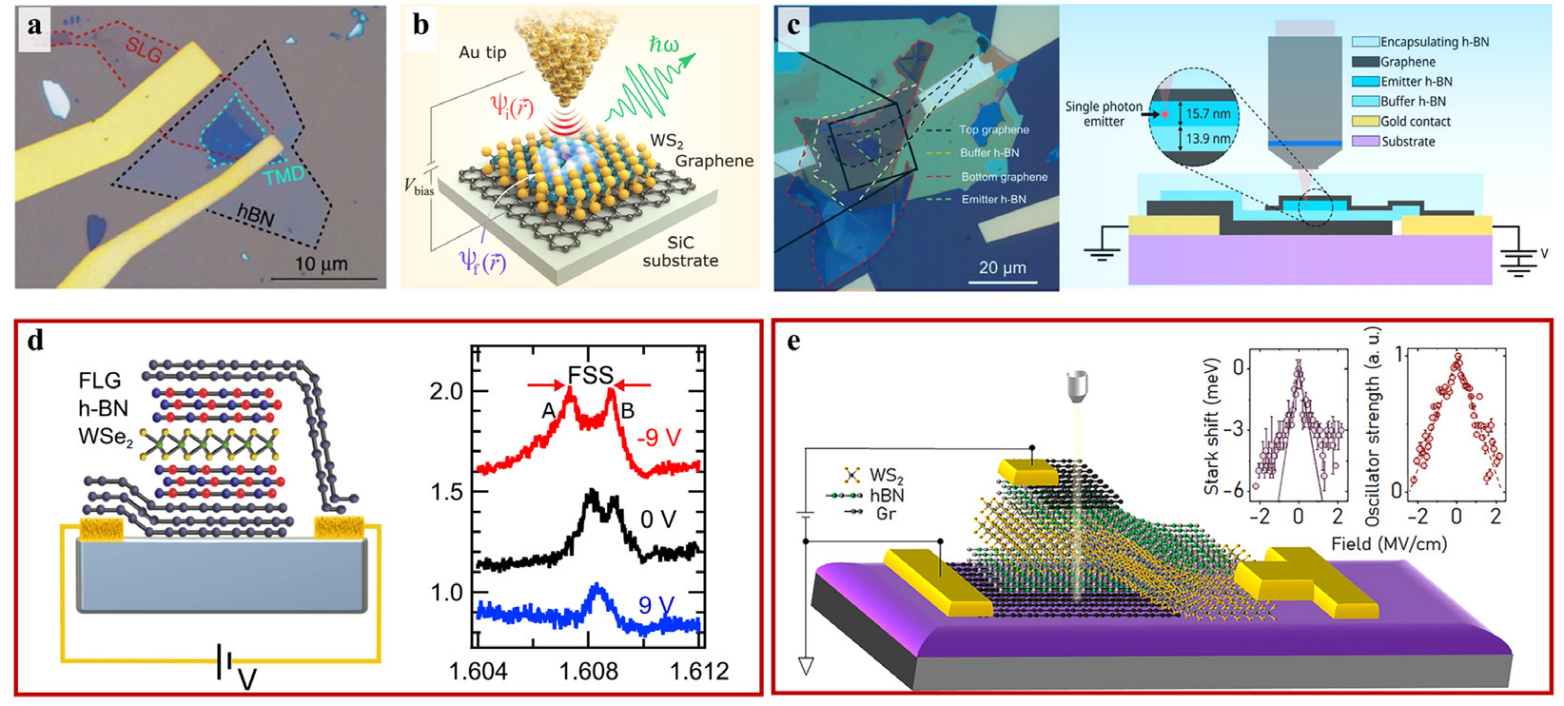
**Electrical modulation of color centers in 2D vdW materials.** (a) Optical microscope image of LED associated with a vertical TMD [106]. (b) Scheme of experimental configuration of tunneling electron-induced photon emission by STM [108]. (c) Schematic of the electrostatic field effect on the h-BN SPE [109]. (d) Schematic device of monolayer WSe_2_ encapsulated in a van der Waals heterostructure with prepatterned electrodes and spectral linecut exhibiting modulation of FSS at the two different voltages [112]. (e) Schematic device of sandwich structure consisted of h-BN, bilayer WS_2_ and graphite layers [113].

Quantum emitters can be electrically excited, which is essential for the scalability and development of on-chip devices. Injecting charge carriers into the defect is one of efficient methods to electrically modulate single photons emissions of 2D materials [105], as illustrated in Fig. 10. For instance, various 2D vdW heterostructures were used to electrically excited SPEs, displaying spectrally narrow electroluminescence (EL) lines of defects similar to that observed under optical excitation [106], [107], [108]. Palacios-Berraquero et al. fabricated a light-emitting diode structure consisting of graphene, thin hexagonal boron nitride and TMD mono- and bi-layers, which enabled all electrical SPEs on a wide spectrum [106], as shown in Fig. 10a. Clark demonstrated electrically excited light emission from single defect by using both lateral and vertical vdW heterostructures. When the current density and temperature were low, they observed thin lines in the EL spectrum that were consistent with optically excited defect-bound excitons. It was demonstrated that the emission comes from single defects in spatially localized regions and has a doublet with the exchange splitting and linearly polarized selection rules [107]. Further, Schuler demonstrated electrical emission of photons from single atomic defects [108], using electron tunneling from a metal tip to chosen discrete defect levels in the WS_2_ bandgap to bring about radiative transitions, as displayed in as shown in Fig. 10b.

Additionally, manipulating the electrostatics of 2D vdW materials has been used to alter characteristics of SPEs, as quantum emitters are responsive to changes in the charge carrier concentration, which impacts the Coulomb interactions on the SPEs. Previous studies have shown that the screening of the localized exciton is the cause of the switching between the on and off states of SPE. Chakraborty et al. identified that atomically thin TMD WSe_2_ is capable of hosting optically active QD-like defects with the internal spin structure [71], which can be modulated by a gate voltage or an external magnetic field. Measuring the fluorescence lifetime of a specific set of quantum dots at two different gate voltages resulted in a factor of four difference. The fluorescence lifetime of a specific set of quantum dots varies by four times when applied two different gate voltages. As shown in Fig. 10c, it was observed that an out-of-plane electrostatic field applied to an emitter in h-BN can reduce the spectral diffusion and Stark tune the emission energy of SPEs, getting close to the lifetime limit of the emitter [109].

Finally, the Stark effect also has been exploited to control the optical and electronic properties of defects in 2D vdW materials by using an external electric field. Producing vdW heterostructures with of h-BN and graphene, Noh et al. demonstrated electrical control of SPE from atomic defects in h-BN [110], owing to the Stark effect. By adding an out-of-plane electric field to graphene gates, they discovered Stark shifts of up to 5.4 nm per GV/m, suggesting the presence of out-of-plane dipole moments related to SPEs from atomic defect. The dependence of Stark shifts on electric filed $\vec{E}$ can be described by the following equation:

(6) $$\Delta \left(\hbar \omega \right)=\;-\;\Delta \vec{\mu }\cdot \vec{E}-\vec{E}\cdot \left(\Delta \vec{\alpha }/2\right)\cdot \vec{E}$$

where ℏ the Plank constant, and ω the photon frequency, $\vec{\mu }$ the dipole moment, and $\vec{\alpha }$ polarizability. After comparing the fitted |Δα| of SPE in h-BN to that measured in diamond $N_{V}$ centers, they found that the electron wave functions in these defects are strongly-bound and have a small volume, as the former is about 2 orders of magnitude smaller than the latter. Further, Scavuzzo et al. observed an asymmetric modulation of both the fluorescence intensity and lifetime in the similar vdW heterostructures by applying the vertical electric field between the negative and positive gate voltage regimes [111]. As shown in Fig. 10d, Chakraborty et al. demonstrated that the WSe_2_ emitters encapsulated in a vdW heterostructure exhibit quantum-confined Stark effect accompanied by a variation in the fine-structure splitting (FSS), due to the anisotropic electron-hole exchange interaction induecd by electric field [112]. The maximum modulation of 1.5 meV in the FSS was achieved in this work. Moreover, they also found that there was a prominent circular polarization of the localized exciton emission when the FSS decreased, confirming the suppression of the anisotropic electron-hole exchange interaction leading to the FSS. As shown in Fig. 10e, Das et al. recently used two different heterostructures composed of layered WS_2_ and graphene to segregate the influences of quantum confined Stark effect and electrostatically induced doping on the excitonic emission in a bilayer WS_2_ [113]. Different from monolayer WS_2_, a bilayer WS_2_ exhibits a linear dependence of the quantum confined Stark effect on electric field, owing to reflection symmetry breaking. In this case, the Stark effect is associated with a significant fluctuation of the exciton oscillator strength because of a partial transformation between intralayer and interlayer excitons.

### 5.2 Integration with photonic structures

**Fig. 11:**
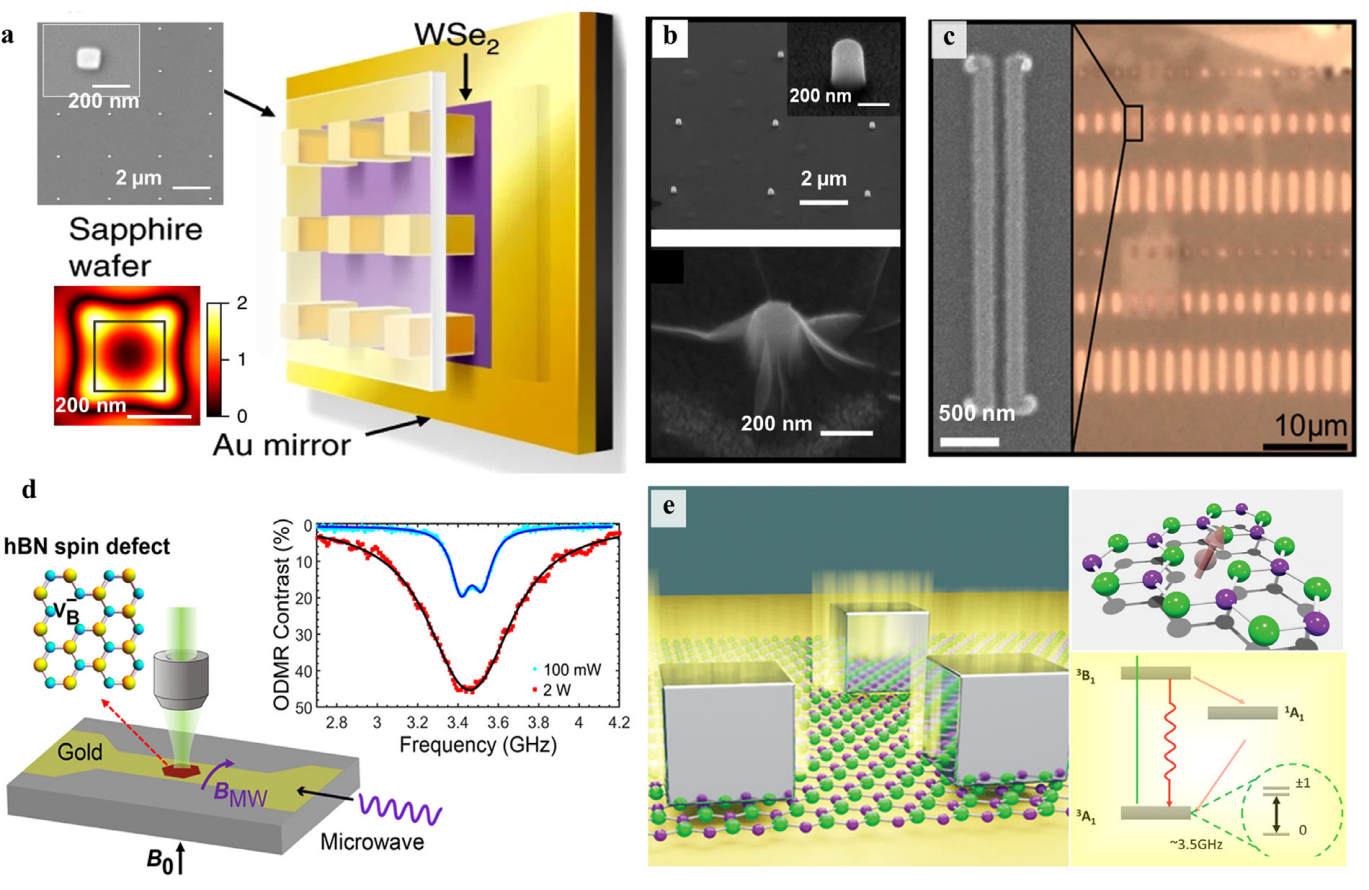
**Integration of color centers in 2D vdW materials into plasmonic structures.** (a) Overview of strain-induced monolayer WSe_2_ excitons coupled to gap modes in a plasmonic Au nanocube cavity array. Upper left: SEM image of the sample. Lower left: simulation showing highest strain at cube corners coincides with plasmonic hot spots [114]. (b) SEM images of a WSe_2_ monolayer covering plasmonic nanopillars [115]. (c) Optical image of the WSe_2_ monolayer flake atop on gold waveguide array. Inset: SEM image of an individual waveguide [117]. (d) Schematic of ODMR measurements for an ion implanted h-BN nanosheet integrated with a gold film microwave strip line [119]. (e) Schematic illustration of coupling $V_{B}^{-}$ centers in FIB irradiated h-BN flakes to a nanocube-on-mirror plasmonic gap cavity [120].

Integrating SPEs with micro- and nano-photonic cavities is another one of efficient strategies for suppressing the spectral diffusion of color centers in 2D vdW materials through a strongly enhanced light−matter interaction. Plasmonic nanostructures, for instance, can generate strong local electromagnetic fields and achieve subwavelength mode confinement, resulting in a significant increase of the excitation and spontaneous emission for the enhancement of the fluorescence of the emitter. The coupling of SPEs to photonic cavities mainly depends on Purcell enhancement of the spontaneous emission rate. The Purcell factor $F_{P}$ can be written as follows:

(7) $$F_{P}\equiv \frac{3}{4\pi^{2}}{\left(\frac{\lambda }{n_{0}}\right)}^{3}\frac{Q}{V_{eff}}$$

where λ the light wavelength, *n_0_* the refractive index at the location of the emitter, *Q* the cavity quality factor, and *V_eff_* is the effective mode volume. The probability of spontaneous emission of a photon placed into the cavity can be calculated with $\beta =\;F_{p}/\left(F_{p}+\;1\right)$, making deterministic photon emission into a single field mode if *F_P_* is sizable to make β≈1. For example, Luo et al. demonstrated a Purcell factor of up to 551 (average of 181) by coupling monolayer WSe_2_ with a metallic nanocube, leading to SPE rates of 42 MHz and a narrow exciton linewidth of 55 µeV. Their work also significantly increased the quantum yields from 1% to 65% (average 44%) [109]. As shown in Fig. 11a-c, plasmon-enhanced SPEs have also been observed in 2D TMD materials using nanorods, nanocones, bowtie antennas and metallic slot waveguides [115], [116], [117]. Similarly, plasmonic arrays of gold and silver nanoparticles also were used to boost the emission of h-BN emitters by a factor of 2, resulting in a quick decrease in its lifetime [118]. A coplanar microwave waveguide composed of a gold film by photolithography was fabricated by Gao et al., realizing a record-high ODMR contrast of 46% along with simultaneous PL enhancement by up to 17-fold from spin defects $V_{B}^{-}$ in h-BN at room temperature (Fig. 11d). It was attributed to the surface plasmon of microwave waveguide of the gold film [119]. Fig. 11d present a scalable approach to dramatically enhance the $V_{B}^{-}$ emission by coupling to a plasmonic gap cavity composed of a gold surface, a silver cube, and few-layer h-BN flakes. With the help of these plasmonic cavities, two orders of magnitude in PL enhancement and a corresponding twofold enhancement of ODMR contrast were obtained [120].

**Fig. 12:**
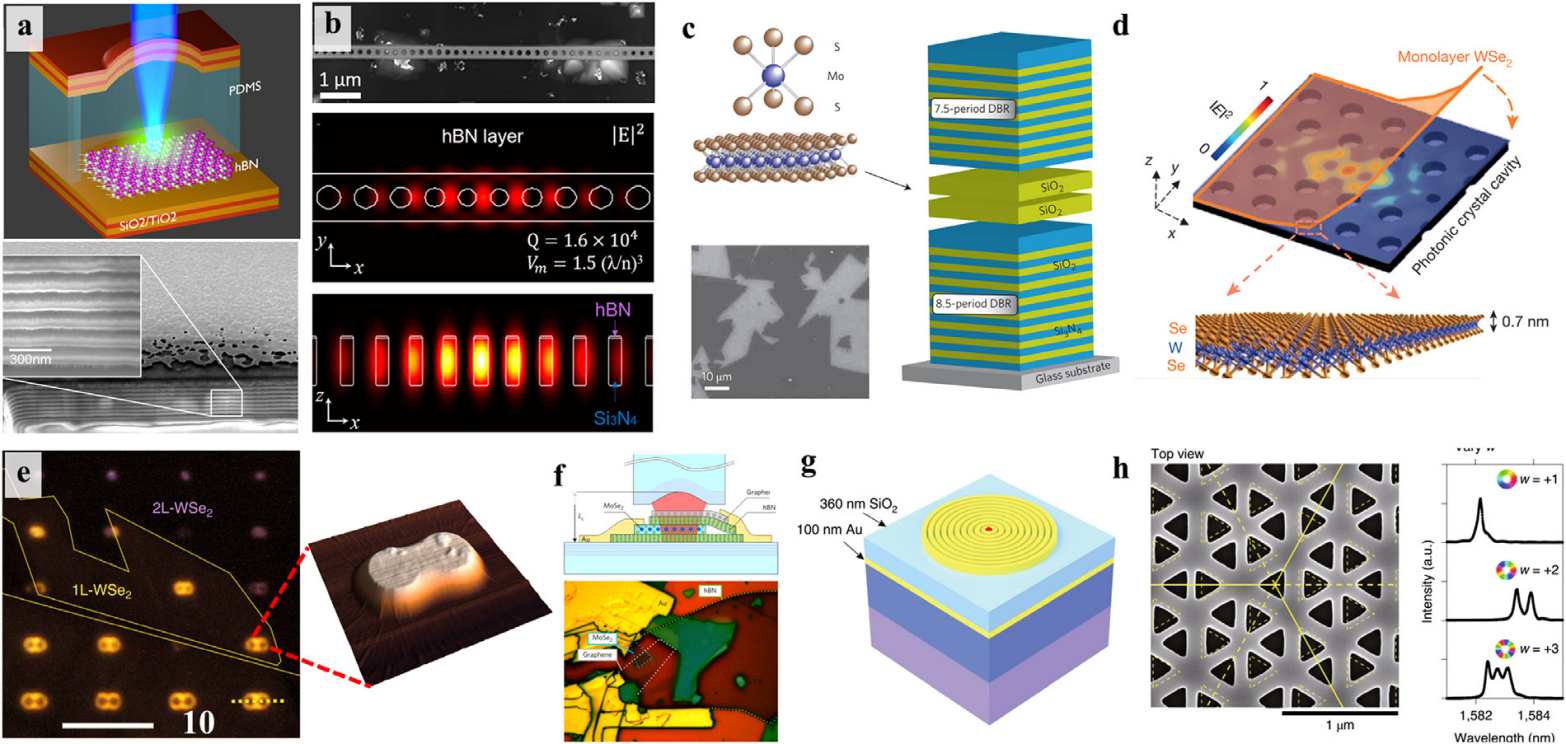
**Integration of color centers in 2D vdW materials into dielectric structures.** (a) Schematic of coupling the h-BN quantum emitter to a microcavity consisting of an etched PDMS spacer sandwiched between hemispherical and flat mirror [121]. (b) Overview of coupling H-BN quantum emitters to PCCs. Top: the SEM image of a 1D photonic crystal circular nanobeam cavity. FDTD simulation of main resonance in h-BN layer for top view (middle) and side view (bottom) [122]. (c) Schematic of the strong light–matter coupling in CVD-grown MoS_2_ by a DBR microcavity [125]. (d) Schematic of hybrid monolayer WSe_2_−PCC lasers with ultralow thresholds [123]. (e) Optical image with unpolarized white light of 1L− and 2L− WSe_2_ on dimer nano-antennas array, respectively [127]. (f) Schematic of coupling a MoSe_2_/h-BN/graphene heterostructure to a fiber cavity [126]. (g) Schematic structure of the elliptical Bragg grating [130]. [h] Experimental research of Dirac-vortex cavities covered with silica. Left: SEM images of a cavity illustrating the C_3v_ symmetry and optical spectra. Right: far fields of cavity modes with different winding numbers [132].

In addition to plasmonic cavities, other dielectric cavity designs have also been explored for coupling with SPEs in 2D vdW materials, as displayed in Fig. 12. Unlike metal used for plasmonic structures, dielectric materials typically have lower losses, enabling efficient Purcell enhancement via high Q factors. As shown in Fig. 12a, Vogl et al. integrated an h-BN quantum emitter into an optical microcavity consisting of an etched PDMS spacer sandwiched between hemispherical and flat mirror. This configuration exhibits a small mode volume of the order of $\lambda^{3}$, enabling Purcell enhancing fluorescence and shortening the excited state lifetime [121]. As a result, this cavity significantly narrowed the spectrum from 5.76 to 0.224 nm and improved the single-photon purity by suppressing off-resonant noise. Photonic crystal cavities (PCCs) made of dielectric silicon nitride were also used to couple with h-BN SPEs [122], resulting in 6-fold PL enhancement of h-BN SPE at room temperature due to a high Q of 3300 (Fig. 12b).

As shown in Fig. 12d, the initial integration of TMDs into a dielectric cavity was impressive, as it leads to the realization of low-threshold lasing from TMD monolayers positioned on the top of a PCC [123]. Continuous-wave nanolasers operating in the visible spectrum were created with an optical pumping threshold of only 27 nanowatts at 130 K, which is comparable to the threshold of PCC lasers. Strong coupling was also demonstrated in a series of studies of exciton-polaritons and polarons using monolayer MoS_2_ and MoSe_2_ integrated into distributed Bragg reflector (DBR) microcavities, characterized by a large Rabi splitting (tens of meV) at room temperature [124], [125], [126]. Theoretically, the polariton dispersion can be expressed using a coupled oscillator model:

(8) $$\left(\begin{array}{cc} E_{cav}+i\hbar \Gamma_{cav} & V_{A}\\ V_{A} & E_{ex}+i\hbar \Gamma_{ex} \end{array}\right)\left(\begin{array}{c} a\\ b \end{array}\right)=E\left(\begin{array}{c} a\\ b \end{array}\right)$$

where *V_A_* the exciton–photon interaction potential, *E_cav_* (*Γ_cav_*) and *E_ex_* (*Γ_ex_*) are the energies (decay rates) of cavity resonant modes and TMD excitons, respectively. *E* is defined as the eigenvalues corresponding to the energies of the polariton modes. The eigenvectors are able to be constructed by the elements a and b, which represent the weighting coefficients of the cavity photon and TMDs exciton for each polariton state, where ${\left|a\right|}^{2}+{\left|b\right|}^{2}\;=\;1$. The eigenvalues are given by the following formula:

(9) $$\begin{array}{ccc} E & = & \frac{E_{ex}+E_{cav}}{2}+\frac{i\left(\hbar \Gamma_{ex}\;+\hbar \Gamma_{cav}\right)}{2}\\ & & \pm \sqrt{V_{A}^{2}-1/4{\left(E_{ex}-E_{cav}+\;i\hbar \Gamma_{ex}-i\hbar \Gamma_{cav}\right)}^{2}} \end{array}$$

where $\hbar \Omega_{Rabi}=\sqrt{V_{A}^{2}+1/4{\left(\hbar \Gamma_{ex}-\hbar \Gamma_{cav}\right)}^{2}}$ is the Rabi splitting at *E_ex_* = *E_cav_*.

Furthermore, dielectric nanoantennas with high refractive index, capable of confining optical modes to ultra-small volumes, were fabricated to enhance ensemble excitonic emissions in TMD WSe_2_. Recently, SPEs were successfully demonstrated in TMD WSe_2_, where the coupled emitters showed emission enhancement of up to $10^{4}$ and averaged increase in the quantum efficiency by a factor of 5, allowing for optical coherence measurements at low excitation power [127], [128], [129]. This PL enhancement can be not only attributed to Purcell enhancement effect but also two other factors: the ratio of the excitation rates and the improved collection efficiency [127]. The effective enhancement factor ${\langle EF\rangle }_{eff}$ can be defined as the product of these three factors. Finally, recent investigations have focused on cavities with distinct features such as polarized cavities, chiral cavities and topological cavities [130], [131], [132]. These cavities possess the unique electromagnetic field properties that can be manifested in the hybrid atom-photon states. Topological cavities, in particular, can generate novel optical modes such bound edge states and chiral vortex states, providing new opportunities to engineer SPEs in 2D vdW materials with high freedom and robustness. In addition, the introduction of topological concept offers new possibilities for combining the optical nonlinearities and quantum optics, leading to the emergence of new collective excitation phenomena and novel optical quantum effects.

## 6 Outlook and conclusion

Promoting the practical development of quantum information technology requires accelerating the reduction of error rates in quantum computing to the lowest possible level, or to proposing entirely new quantum schemes. In order to achieve these goals, major advances in materials science and engineering along with new fabrication and synthesis techniques, will be critical breakthroughs. Additionally, the development of other quantum applications such as quantum sensing and communication devices also drive progress in this field. In the last section, we discuss some promising directions for further research on these two points.

Material design. Experimentally examining various point defects in a range of host materials remains a continuing challenge. Highly predictive theoretical investigations can significantly accelerate the discovery of new candidates by initially identifying target defects that can be examined experimentally. The first inevitable step is to calculate the electronic structure of point defects for this purpose. DFT-based first-principles calculations are widely recognized for providing comprehensive insights into the physics and chemistry of point defects, as well as their impact on structural, electronic and optical characteristics. Theoretical approaches have proven effective in understanding the results of experimental results related to defects, such as magneto-optical experiments, as they often provide indirect information about the microscopic characteristics of the defects. However, it is necessary to explore new methods for rapid screening and calculations to identify properties of suitable defect qubits, as ab initio methods can be time-consuming. A new computing frame for discovering new host materials has been proposed, utilizing high-throughput screening of the ZPL to identify atom-like defect qubits. Janak's theorem can be used to quickly check for the errors in ZPL, and the standard method can reduce the number of iterations needed for excited state calculations compared to the default procedure [133]. In addition, cluster correlation expansion (CCE) techniques have been proposed as a prominent tool to calculate the $T_{2}$ of defect spins in these host materials with high accuracy. Based on CCE, Kanai et al. calculated more than 12,000 host materials at natural isotopic abundance and suggest potential host ones with long $T_{2}$ up to 47 ms and provides an opportunity to explore promising functional materials for quantum applications [80]. Finally, machine learning (ML) has emerged as a novel and distinctive method for material design and prediction of properties. Frey et al. recently developed an approach to swiftly investigate primary characteristics of point defects in 2D vdW materials, combining deep transfer learning, machine learning, and first-principles calculations [134]. The physics-informed featurization was utilized in this study to create a concise description of defective structures and provide a universal image of defects spanning materials systems. They almost built 10,000 defective structures from more than 150 wide band gap semiconductors and layered metal chalcogenides. By utilizing their calculations, they were able to assess ensemble machine learning models based on physical featurization, examining band structures and formation energies of more than 1000 defects. These models have advantages including no requirement for electronic structure calculations and the usage of easily accessible descriptors, encoding information about local relaxation and electronic interactions that manifests defect physics.

Quantum application. Optically addressable quantum defects have attracted extensive interest and find extensive applications in quantum sensing. Their main advantages include fast dynamics, non-destructive and label-free operation, and high sensitivity from the atom-like nature of quantum systems, combined with the high spatial resolution. However, gaining control over a sensor surface is one of critical challenges in using quantum defects as nanosensors. In order to achieve high sensitivity and resolution, quantum defects need to be positioned close to the sensing target, which requires proximity to the host surface. Interestingly, atomically thin 2D vdW materials offer a solution as defects on these materials could be positioned just a few angstroms close to the sensing object, significantly enhancing their sensitivity. In addition, their capability for direct integration into nanoparticles make defect qubits of 2D vdW materials especially attractive as in situ sensors. However, the limited wavelength range may be an obstacle for quantum sensing with defects of 2D vdW materials. This would drive the search for new defects in 2D vdW materials with emissions covering different bands of the electromagnetic spectrum, especially in the telecom band (∼1500 nm). On the other hand, the capability to serve as a PSI makes quantum defects particularly suitable for diverse quantum applications. The spin degree of freedom can be used as a local quantum memory, whereas the photon can function as a flying qubit. Integrating quantum defects with specially designed photonic nanostructures or nanocavities to enhance the atom–photon interaction is one major frontier in the research field of quantum information science. Previous studies have dedicated great efforts on integrating color centers into nanophotonic structures, but the sensitivity to fluctuation of charges for the optical behaviors hinders high cooperativity in these structures. Future work in this field is able to greatly benefit from 2D vdW materials, due to the easy formation of high-quality heteroepitaxial structures. Finally, an appealing direction is to integrate defect spins with on-chip nanophotonics, thereby discovering on-demand quantum sensors, as well as miniaturized information processing units. An on-chip integrated package based on micro/nano-fabricated photonic units, including metalenses, couplers, waveguides, and nanosensors, could dramatically reduce optical losses, as well as abandon traditional large-sized free-space optics.

## Declaration of competing interest

The authors declare that they have no conflicts of interest in this work.
